# Diversity and Structure of the Endophytic Bacterial Communities Associated With Three Terrestrial Orchid Species as Revealed by 16S rRNA Gene Metabarcoding

**DOI:** 10.3389/fmicb.2020.604964

**Published:** 2020-12-15

**Authors:** Pasquale Alibrandi, Sylvia Schnell, Silvia Perotto, Massimiliano Cardinale

**Affiliations:** ^1^Department of Life Sciences and Systems Biology, University of Turin, Turin, Italy; ^2^Institute of Applied Microbiology, Justus-Liebig-University Giessen, Giessen, Germany; ^3^Department of Biological and Environmental Sciences and Technologies, University of Salento, Lecce, Italy

**Keywords:** bacterial endophytes, orchids (Orchidaceae), microbiota, metabarcoding analysis, seeds

## Abstract

The endophytic microbiota can establish mutualistic or commensalistic interactions within the host plant tissues. We investigated the bacterial endophytic microbiota in three species of Mediterranean orchids (*Neottia ovata*, *Serapias vomeracea*, and *Spiranthes spiralis*) by metabarcoding of the 16S rRNA gene. We examined whether the different orchid species and organs, both underground and aboveground, influenced the endophytic bacterial communities. A total of 1,930 operational taxonomic units (OTUs) were obtained, mainly Proteobacteria and Actinobacteria, whose distribution model indicated that the plant organ was the main determinant of the bacterial community structure. The co-occurrence network was not modular, suggesting a relative homogeneity of the microbiota between both plant species and organs. Moreover, the decrease in species richness and diversity in the aerial vegetative organs may indicate a filtering effect by the host plant. We identified four hub OTUs, three of them already reported as plant-associated taxa (*Pseudoxanthomonas, Rhizobium*, and *Mitsuaria*), whereas *Thermus* was an unusual member of the plant microbiota. Core microbiota analysis revealed a selective and systemic ascent of bacterial communities from the vegetative to the reproductive organs. The core microbiota was also maintained in the *S. spiralis* seeds, suggesting a potential vertical transfer of the microbiota. Surprisingly, some *S. spiralis* seed samples displayed a very rich endophytic microbiota, with a large number of OTUs shared with the roots, a situation that may lead to a putative restoring process of the root-associated microbiota in the progeny. Our results indicate that the bacterial community has adapted to colonize the orchid organs selectively and systemically, suggesting an active involvement in the orchid holobiont.

## Introduction

Plants in natural and agricultural ecosystems interact with a multitude of microorganisms that colonize both the internal tissues and the outer surfaces of both underground and aboveground plant organs (Compant et al., 2019; Escudero-Martinez and Bulgarelli, 2019). These plant-associated microbial communities are collectively known as the plant microbiota (Marchesi and Ravel, 2015) and can theoretically comprise mutualistic, commensal and latent pathogenic strains in apparently healthy plants (Brader et al., 2017). Bacteria and fungi, the dominant components of the plant microbiota, can deeply influence plant growth and responses to stresses. For these reasons, several studies in the last decade focused on the structure and functions of the plant microbiota, with the aim to link them to plant fitness and crop productivity (Bulgarelli et al., 2013; Compant et al., 2019; Tian et al., 2020).

Endophytes can be defined as components of the plant microbiota that colonize internal healthy plant tissues and establish non-harmful relationships with their host (Hardoim et al., 2015). This definition includes mutualistic endophytes that form recognizable structures during plant colonization - such as mycorrhizal fungi in the roots of land plants and rhizobia in legume root nodules - whose interactions with the host plant have been extensively investigated because of their beneficial influence on plant growth, productivity and health (Das et al., 2017; Masson-Boivin and Sachs, 2018; Genre et al., 2020). However, there is ample evidence that many apparently commensalistic endophytes can also promote plant growth and defense (Santoyo et al., 2016; Hossain et al., 2017), but the ecology and functions of these beneficial endophytes is less understood. In particular, plant growth-promoting (PGP) endophytic bacteria can promote plant growth by mechanisms that include the release of phytohormones (Patten and Glick, 2002; Glick, 2012, 1995; Rashid et al., 2012; Duca et al., 2014), nitrogen fixation (Govindarajan et al., 2008; Urquiaga et al., 2012), improved acquisition of mineral nutrients (Malinowski et al., 2000; Rahman et al., 2005), production of growth-promoting compounds (Glick et al., 1998; Taghavi et al., 2009; Kumar et al., 2019) and increased stress tolerance (Kohler et al., 2008). Furthermore, part of the bacterial endophytic community showed tolerance toward heavy metals and pollutants, thus finding an important application for bioremediation (Guo et al., 2010; Chen et al., 2012; Stępniewska and Kuźniar, 2013). Bacterial endophytes can also protect the host plant against pathogen invasion and disease through direct biocontrol activities or by inducing plant systemic resistance (Berg and Koskella, 2018; de Vrieze et al., 2018).

The fact that only a minority of the environmental bacteria can be cultivated in the laboratory has been a limiting factor for environmental microbiologists, restricting knowledge about the interaction mechanisms between the microbiota and the plant. Next generation sequencing (NGS) technologies have allowed large-scale studies independent from the cultivation approach, providing details on taxonomic structure, abundance and potential physiological features of the microbial endophytes associated with plants (Knief, 2014). By using these molecular tools, it is possible to estimate the evolution and adaptation of the microbes associated with the host and to understand how they are related to each other (Khare et al., 2018; Lucaciu et al., 2019).

Orchidaceae is one of the largest plant families on Earth (Christenhusz and Byng, 2016). Orchids grow in a wide range of habitats and include epiphytes as well as terrestrial species. Most orchids are photosynthetic at adulthood, but about 200 species are fully achlorophyllous and rely on their mycorrhizal fungal partners for the provision of carbon (Hynson et al., 2013). Several forest orchid species develop green leaves but remain at least partly dependent on mycorrhizal fungi for carbon supply (Selosse and Roy, 2009). Irrespective of their trophic strategies, orchids are highly dependent on the relationship with mycorrhizal fungi, and their distribution is often correlated with the distribution of their mycorrhizal fungal partners (McCormick et al., 2018). Because of their importance in the survival of these endangered plants, most of the microbiological investigations on orchids have focused on their mycorrhizal associations, leaving aside the potential role of other microbial partners. Bacterial endophytes in orchids have been mainly investigated in commercially valuable species by isolation after surface sterilization of roots and/or leaves, and many isolates have been found to both increase seed germination and promote plant growth (Tsavkelova et al., 2007; Faria et al., 2013; da Silva et al., 2015; Gontijo et al., 2018; Herrera et al., 2020). By contrast, culture-independent approaches have been rarely applied to investigate the endophytic bacteria of orchids, and data are only available for the epiphytic orchids *Dendrobium officinale* and *Dendrobium catenatum* (Yu et al., 2013; Li et al., 2017). In these orchid species, endophytic bacteria belonging mainly to Proteobacteria were identified by means of PCR-DGGE and pyrosequencing, showing diversity in the roots and stems. In addition, meta-*nifH* sequencing suggested the presence of common diazotrophs in several *D. catenatum* tissues, which could play an important role in nitrogen supply (Li et al., 2017). Here, we have explored the endophytic bacterial communities of three terrestrial orchid species, namely *Neottia ovata* (L.) Bluff & Fingerh, *Serapias vomeracea* (Burm. F.) Briq. and *Spiranthes spiralis* (L.) Chevall. We investigated different plant organs in order to characterize the taxonomic diversity and the structure of the associated microbiota, and to identify a core microbiota. The same three orchid species were already found to host endophytes with a notable plant growth promoting potential, as assessed by cultivation and characterization of bacterial isolates (Alibrandi et al., 2020). Based on results on other plant species (Bulgarelli et al., 2013; Haruna et al., 2018), our aim was to verify the following hypotheses in orchids: (i) endophytic bacterial communities create distinctive phylogenetic structures in the various plant organs, and (ii) the reproductive organs (seeds) represent a reservoir of endophytes that can be vertically transmitted. To test these hypotheses, two comparisons were made: first, we compared the bacterial microbiota associated with different plant organs (roots, leaves, stems and capsules) of the three orchid species; second, we included the seed microbiota of *S. spiralis* (the only species for which seeds were available at the time of sampling) to analyze the distribution of unique, shared and core OTUs within this species.

This study provides new insights into the distribution of bacteria across different organs of terrestrial orchid species, and sheds light on the fraction of the bacterial community potentially transferred to the next plant generation by vertical transmission, pointing out a possible role of bacterial endophytes in orchid conservation.

## Materials and Methods

### Orchid Sampling and Surface-Sterilization

The plant species investigated in this work were *Neottia ovata* (L.) Bluff & Fingerh., *Serapias vomeracea* (Burm. f.) Briq. and *Spiranthes spiralis* (L.) Chevall., three Mediterranean orchid species that grow naturally in Italy. Samples of roots, stems, leaves and capsules of the three different orchids were harvested during flowering in the Liguria region (Italy), stored on ice in sterile containers, transported to the laboratory and kept at 4°C before being processed (within 2 days). Furthermore, seeds from surface sterilized capsules (see below for sterilization) of *S. spiralis* were also analyzed.

Plant samples from aboveground organs (stems, leaves and capsules) were surface–sterilized by stepwise immersion in 70% ethanol for 1 min, then in 2.5% sodium hypochlorite for 2 min and finally in 70% ethanol for 1 min, followed by five rinses in sterile distilled water (Alibrandi et al., 2018). Root samples were thoroughly rinsed with sterile water, sonicated and surface-sterilized with 95% ethanol for 20 s followed by immersion in 5% sodium hypochlorite for 3 min and washed seven times with sterile distilled water. Furthermore, we obtained the seeds of *S. spiralis* from surface-sterilized capsules. The surface-sterilized capsules were placed into a sterile falcon tube, sealed and seeds were recovered from the tube bottom once the capsules hatched.

Five biological replicates, each collected from different plant individuals, were used for each orchid organ, for a total of 65 samples. Samples were named according to the orchid species (NO for *N. ovata*, SV for *S. vomeracea* and SS for *S. spiralis)*, and organs (R for root, L for leaf, St for stem, C for capsule, S for seed), the numbers indicating the biological replicate (Table 1).

**TABLE 1:** Samples of the orchid species/organs investigated in this study.

Sample name	Orchid species	Organ
(1–5)NO.pna.R	*Neottia ovata*	Root
(1–5)NO.pna.L	*Neottia ovata*	Leaf
(1–5)NO.pna.St	*Neottia ovata*	Stem
(1–5)NO.pna.C	*Neottia ovata*	Capsule
(1–5)SV.pna.R	*Serapias vomeracea*	Root
(1–5)SV.pna.L	*Serapias vomeracea*	Leaf
(1–5)SV.pna.St	*Serapias vomeracea*	Stem
(1–5)SV.pna.C	*Serapias vomeracea*	Capsule
(1–5)SS.pna.R	*Spiranthes spiralis*	Root
(1–5)SS.pna.L	*Spiranthes spiralis*	Leaf
(1–5)SS.pna.St	*Spiranthes spiralis*	Stem
(1–5)SS.pna.C	*Spiranthes spiralis*	Capsule
(1–5)SS.pna.S	*Spiranthes spiralis*	Seed

*For each sample, five biological replicates (collected from different plant individuals) were analyzed.*

### DNA Extraction

Total DNA was extracted separately from each organ sample (roots, leaves, stems, capsules and seeds) of each orchid species using the Qiagen DNeasy kit (QIAGEN^®^,Hilden, Germany) according to the manufacturer’s instructions. The resulting 65 DNA samples were checked for integrity by electrophoresis in a 1% agarose gel and stored at −20°C.

### PNA Clamps

The endophytic bacterial microbiota was investigated by 16S rRNA gene metabarcoding (V3 – V4 regions). However, the molecular characterization of the microbiota inside host plant tissues was conditioned by the co-amplification of mitochondrial and plastid rRNA genes extracted from the eukaryotic plant cells. To reduce this issue, a suitable approach is the use of sequence-specific peptide nucleic acid (PNA) clamps, which bind to, and block, the amplification of host-derived DNA (Lundberg et al., 2013). Although there are universal PNAs available to block plant mitochondrial (mPNA) and plastid (pPNA) sequences, unfortunately they are not effective on a large number of plant species. In order to select the PNAs that could bind to the greatest number of contaminating sequences, an *in silico* analysis of mitochondrial and plastid sequences obtained from a preliminary sequencing was carried out. Several PNA clamps already tested on some plant species have been evaluated, and the best result *in silico* was obtained using the universal probe pPNA (5′-GGCTCAACCCTGGACAG-3′) according to Lundberg et al. (2013), and a newly designed mPNA probe (5′-CTACCGACGCTGGGG-3′), respectively, for plastid and mitochondrial sequences. To verify the effective reduction of host-derived sequences, four samples from root, stem, leaf and capsules of *S. spiralis* were amplified with or without the PNA clamps: the effect of PNA clamps on the relative abundance of bacterial, plastid and mitochondrial sequence reads was assessed by Student’s *t*-test using SPSS 20 (IBM Corporation, Armonk, NY, United Sates).

### Library Preparation and Ion Torrent Sequencing of the Bacterial 16S rRNA Gene

The DNA obtained from all samples was used as template for the metabarcoding of the 16 rRNA gene by Ion Torrent sequencing. The hypervariable V4 and V5 regions of the 16S rRNA genes were amplified by PCR using the primers 520 F (5′-AYTGGGYDTAAAGNG-3′) and 907 R (5′-CCGTCAATTCMTTTRAGTTT-3′) (Claesson et al., 2009; Engelbrektson et al., 2010). Two PCR reactions were performed. In the first reaction, the DNA of all the samples was amplified including mPNA and pPNA clamps to reduce co-amplification of host plant DNA. PCR was carried out at a final volume of 15 μl including the following reagents: 10 ng of template DNA, 3 μl of 5x KAPAHiFi (KAPABiosystems, Woburn, MA, United States) buffer, KAPA dNTP mix 200 μM each, 5 pmol of each primer each and KAPAHiFi polymerase 0.3 units. The following PCR program was used: 3 min 95° C, followed by 35 cycles of (20 s 95°C – denaturation; 15 s 78°C – PNA annealing; 30 s 55°C – primer annealing; 30 s 72°C – elongation) and 5 min at 72°C. PCR products were visualized by agarose gel electrophoresis, and then used as template for the second PCR reaction. The second PCR was carried out with primer 520 F and 907 R comp, adapter (5′-CCATCTCATCCCT GCGTGTCTCCGACTCAG-3′) and barcodes, with KAPA2G Robust HotStart Ready-Mix (KAPA Biosystems) as described by Ambika Manirajan et al. (2016). Both PCRs were performed with a MyCycler TM (Bio-Rad). The PCR products were purified using QIAquick PCR purification kit (QIAGEN GmbH), and further purified with DNA purification beads NucleoMag NGS clean-up kit (MACHEREY-NAGEL GmbH & Co., KG) to remove contaminants (nucleotides, primers, adapters, enzymes, buffer additives, and salts). Amplicon concentration was estimated by measuring the fluorescence with a spectrophotometer. Ion Torrent sequencing was performed on pooled PCR products. Emulsion PCR (Ion PGM Hi-Q View OT2 kit, Life Technologies) was performed with Ion One Touch 2 (Life Technologies, Carlsbad, CA, United States). The quality of the final product was assessed with the Ion Sphere Quality Control Kit (Life Technologies, Carlsbad, CA, United States) and loaded on 318 chip for sequencing (Life Technologies), using an Ion PGM sequencer (Life Technologies, Carlsbad, CA, United States).

The sequences were submitted to the European Nucleotide Archive (ENA, www.ebi.ac.uk/ena) under the accession number PRJEB40289.

### Bioinformatic Analysis of Metabarcoding Sequences

Raw sequence data were processed with QIIME, version 1.9 (Caporaso et al., 2010). Sequences were de-replicated and assigned to specific samples according to the corresponding barcodes, filtered by length and quality (length: 350–450 bp; quality threshold: 20) and grouped in operational taxonomic units (OTU) with uclust (Edgar, 2010) using a 97% identity threshold. Chimeric sequences were removed with Chimera Slayer (against the Silva base reference alignment). Reference sequences for each OTU were assigned to a taxonomic group by comparison with the Silva database (version 132). Singletons and contaminant sequences (mitochondrial and plastid) were eliminated.

To obtain a more accurate classification, phylogenetic trees were built by aligning the 16S rRNA gene sequences of relevant OTUs (such as the hub taxa) to the type strain sequences of the nearest genera, using the Muscle alignment implemented in the megaX software (Kumar et al., 2018). Phylogenetic trees were inferred with the maximum likelihood methods, using RaxML (maximum randomized axillary probability) as implemented in the open source CIPRES Science Gateway^1^. One-thousand bootstrap resamplings were generated, and values above 50% were shown at the branches. The trees were visualized and edited using the open source iTOL (Interactive tree of life^2^).

The composition of the microbiota was compared between the different orchid species and organs analyzed. Taxa relative abundances were calculated on the OTU table without any rarefaction; bar charts were plotted using the ggplott2 package in R software version 1.2.1335 (Gergs and Rothhaupt, 2015; Wickham, 2016).

The data sets were processed after different rarefactions: a single rarefaction (221 reads per sample), based on the sample with the least number of readings, was used for the comparison of the alpha diversity between the three orchid species, while three rarefactions were used for the comparison of organs within each species (2965, 1617, and 221 reads per sample for *N. ovata*, *S. vomeracea* and *S. spiralis*, respectively). Community richness (“observed OTUs” index), diversity (Shannon index) and phylogenetic diversity (PD index) were calculated using the QIIME script alpha_diversity.py; the R statistical software was used to perform ANOVA with Tukey HSD *post hoc* test (*p* < 0.05).

Differences in community structure between species and organs were determined by permutational multivariate ANOVA on Bray-Curtis similarity matrices after 9,999 permutations and plotting (Non-metric multidimensional scaling – NMDS – ordination). Analyses were performed using the Qiime script beta_diversity.py. NMDS and boxplots were calculated in R software version 1.2.1335 with the vegdist function in “vegan” and “ggplot2” packages, respectively. Differences at *p* < 0.05 were regarded as statistically significant.

### Shared and Core OTUs

Recent studies (Bulgarelli et al., 2012; Lundberg et al., 2012) have defined a core range of species of the microbiota associated with plant species as taxonomic core microbiota. Bacterial OTUs shared between the different orchid species and between the different organs of each species were identified. In this work, the OTUs occurring in 100% of the species or organs were considered as belonging to the core microbiota, as calculated with the QIIME script compute_core_microbiome.py. This was considered as the “stable” fraction of the community suggesting possible key functional roles for the host. For visualization and interpretation of these data, Venn diagrams were generated using the free online tool of the Ghent University^3^.

### Co-occurrence Network Analysis

To identify hub OTUs, a co-occurrence analysis was performed with the Co-occurrence Network Inference software (CoNet; Faust and Raes, 2016), using not-normalized data, as recommended, to reduce the compositional effect (Berry and Widder, 2014). To ensure statistical robustness, only OTUs with more than 50 reads and occurring in at least 20 samples were considered. Pairwise scores were calculated for four measures: Bray-Curtis and Kullback–Leibler similarities, and Pearson and Spearman correlations. One-hundred permutations (with row shuffling re-sampling and re-normalization for correlations), and 100 bootstrap scores, were generated. Unstable edges (outside the 2.5–97.5 percentiles of the bootstrap distribution) were eliminated. The individual *p*-values of the four measures were merged with the Brown’s method. Only edges with false discovery rate (FDR)-corrected *p*-values < 0.05 were retained, and only if they were supported by at least three of the four measures. The network layout was generated with the “edge-forced spring embedded” algorithm, which leads to unbiased networks showing interconnected nodes closer to each other and less-linked ones placed in the outside position. Network legends were created with the Cytoscape Add-on “Legend creator^4^”. Hub taxa were identified by scoring the degree (number of connections) against the betweenness centrality and the closeness centrality, according to Agler et al. (2016). The taxonomical identity of the hub OTUs was confirmed/improved by manual BLAST.

## Results

### Sequencing Output, OTU Clustering, and Effect of PNA Blockers

Ion Torrent sequencing generated 6,460,549 raw sequences. After length and quality filtering, 4,117,638 sequences remained, which were grouped into OTUs at 97% similarity level. The mitochondrial and plastid sequences and the singletons were removed to end up with 1,469,200 total sequences (Supplementary Table 1) grouped in 1,930 OTUs. Two samples (both corresponding to leaves of *S. spiralis*) were eliminated due to the low number of reads (Supplementary Table 1).

**FIGURE 1:**
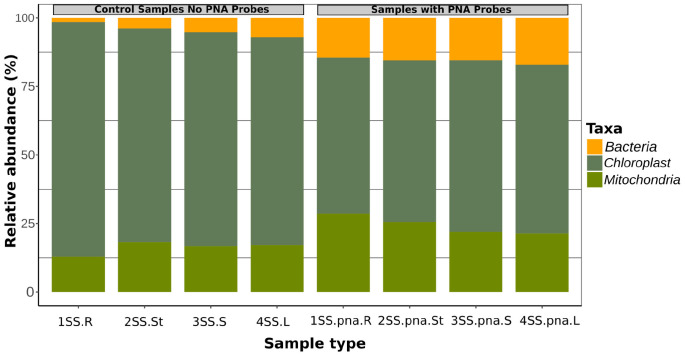
Relative fraction of sequences belonging to Bacteria, Plastids and Mitochondria in four orchid samples, as obtained by metabarcoding of 16S rRNA genes without PNA blockers, compared with the same samples with PNA.

The addition of the PNA probes significantly reduced the relative abundance of plastid sequences with respect to the same samples without PNA probes (*t*-test, *p* = 0.0095), while mitochondrial sequences were slightly but not significantly higher (*p* = 0.052) (Figure 1). Relative abundance of bacterial sequences was significantly increased in the samples amplified with PNA probes (*t*-test, *p* = 0.00048; Figure 1).

### Composition of the Bacterial Orchid Microbiota

**FIGURE 2:**
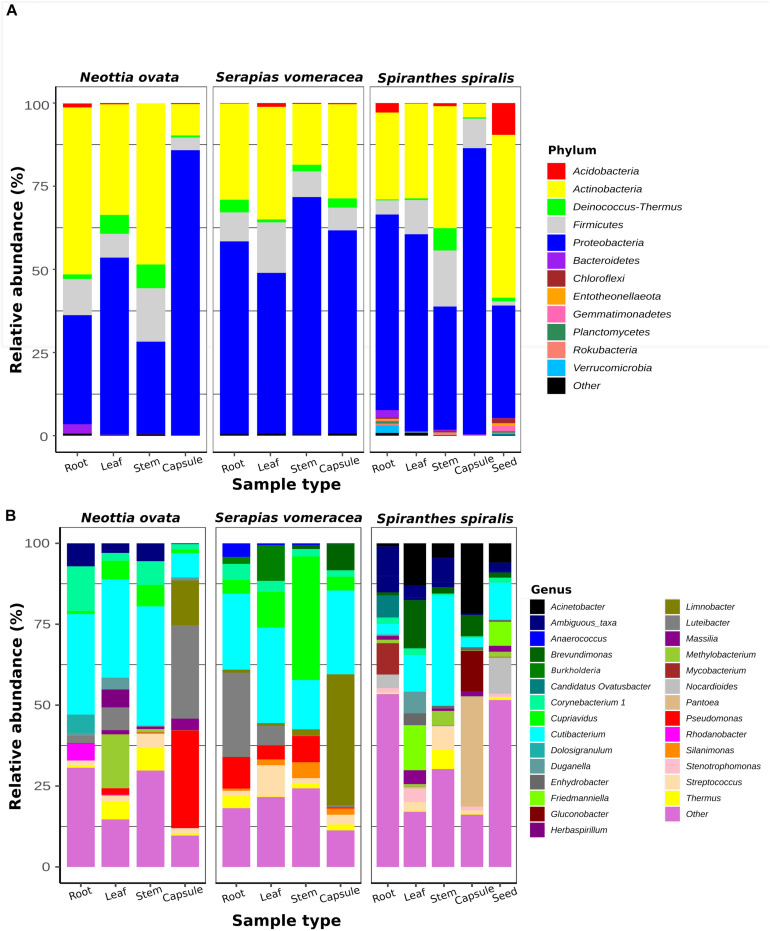
Bacterial taxa distribution in the orchid microbiota. Relative abundance of panel **(A)** predominant bacterial phyla (>1%) and **(B)** predominant genera (>0.5%), as obtained by 16S rRNA gene metabarcoding of orchid plants (*n* = 5).

To identify the taxa associated with the orchid samples and to analyze their distribution in the different species and organs, two taxonomic levels (phylum and genus) were considered (Figure 2). The two most represented phyla were Proteobacteria and Actinobacteria, with Proteobacteria dominating (from 37 to 86%) in most orchid samples. Proteobacteria were particularly abundant in the *S. vomeracea* stems (71.40%) and in the capsules of *N. ovata*, *S. vomeracea*, and *S. spiralis* (85.72, 61.01, and 86.03%, respectively). Complementarily, Actinobacteria were dominant in *N. ovata* roots and stems (50.02 and 48.47%) and in *S. spiralis* seeds (48.95%). Firmicutes (from 1.26 to 16.83%) and Deinococcus-Thermus (from 0.29 to 7.14%) were less represented but distributed in all organs and species (Figure 2A). By contrast, Acidobacteria were rather infrequent, except in the *S. spiralis* seeds (9.59%) (Figure 2A).

Among genera, *Cutibacterium* was distributed in all samples (Figure 2B). This genus was abundant in the roots (31.02%), stems (36.83%) and leaves (30.40%) of *N. ovata*, and less represented in the capsules, in which *Pseudomonas* (30.18%) and *Luteibacter* (28.77%) were the dominant genera.

**FIGURE 3:**
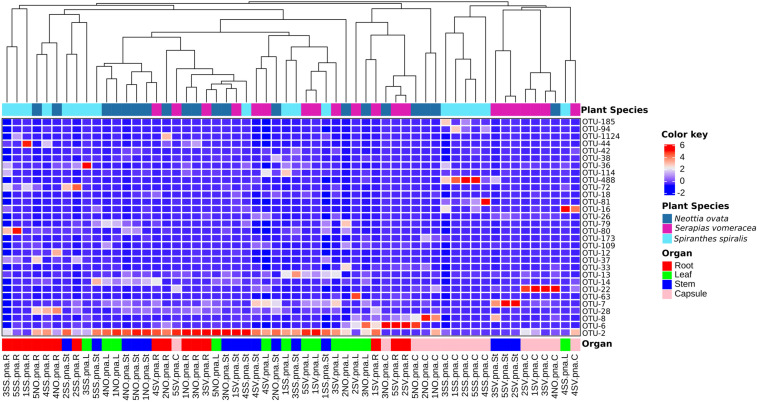
Heatmap of major bacterial OTUs (>0.5%) in the orchid microbiota. Samples were clustered by orchid species (top row) or organs (bottom row). Legends to the right of the figures indicate color scheme for log transformed OTU abundance, species and organ samples.

In *S. vomeracea*, *Luteibacter* (26.08% in the root), *Cupriavidus* (38.20% in the stem), *Cutibacterium* (29.50% in the leaf) and *Limnobacter* (40.53% in the capsule) were the most abundant genera. In *S. spiralis*, the distribution model mainly concerned *Pantoea* and *Acinetobacter* (33.83 and 21.79% in the capsule) and *Cutibacterium* (34.00% in the stem), while in leaves there was no clear distribution of dominant genera (Figure 2B). All minor genera (<0.5%) were mostly distributed in both roots and seed of *S. spiralis* (Figure 2B, indicated as “other”). The distribution and abundance of major OTUs (>0.5% of the total orchid microbiota) were used to create a heat map. The dendrogram resulting from single hierarchical clustering showed only a partial tendency of the samples to group by organ and/or species (Figure 3).

### Alpha- and Beta-Diversity

**FIGURE 4:**
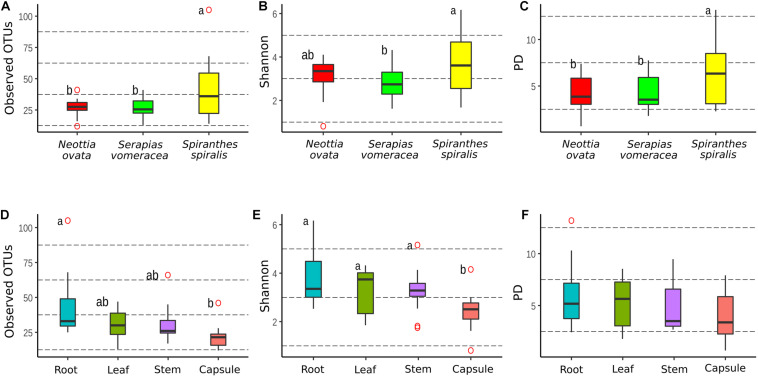
Comparison of alpha-diversity metrics between the microbiota of three Mediterranean orchid species. Richness (observed OTUs), diversity (Shannon) and phylogenetic diversity (PD) for different orchid species **(A–C)** and organs **(D–F)**. ° indicates outliers. Different letters above the bars indicate significantly different means (Tukey test, *p* < 0.05); no letters (panel **F**) indicate no statistically significant differences.

The dataset was processed to obtain three rarefied sub-datasets. A first sub-dataset was rarefied to 221 reads and used to compare the alpha diversity values of the three orchid species and the organs of *S. spiralis*, whereas two more sub-dataset were rarefied to 2,965 and 1,617 reads to compare the organs of *N. ovata* and *S. vomeracea*, respectively. Good’s coverage was >99% for all *N. ovata* samples (Supplementary Table 2), >96% for all *S. vomeracea* samples (Supplementary Table 3), and >85% for all *S. spiralis* samples except two ones with 76 and 82% (Supplementary Table 4). The alpha diversity of the microbiota in each sample was analyzed by considering the Observed OTUs (richness), Shannon (diversity) and Phylogenetic Diversity indices (Supplementary Tables 2–4). The *S. spiralis* microbiota showed the highest values in all indices, when compared with the other two orchid species (Figures 4A–C). When the different organs of the three species were considered, there was a trend of decreasing values in richness, diversity and phylogenetic diversity indices along a vertical progression from roots to capsules, with roots and capsules being significantly different for richness and capsule being significantly different from all other samples for diversity (Figures 4D–E).

Comparison of the same indices in the different organs within each individual orchid species showed some significant differences in *N. ovata* (Supplementary Figures 1A–C) and *S. spiralis* (Supplementary Figures 1G–I), but not in *S. vomeracea* (Supplementary Figures 1D–F). In particular, OTU richness was significantly higher in the root samples of *N. ovata*, as compared to stem and capsule, whereas root and leaf samples were different from capsule for the Shannon index. Finally, root and stem samples of *N. ovata* were significantly different in the phylogenetic diversity index. Root and capsule samples of *S. spiralis* were significantly different in both richness and diversity indices, but not for phylogenetic diversity (Supplementary Figures 1G–I). In particular, both OTU richness and diversity (Shannon index) were significantly higher in the root samples of *S. spiralis*, as compared to the capsules.

**FIGURE 5:**
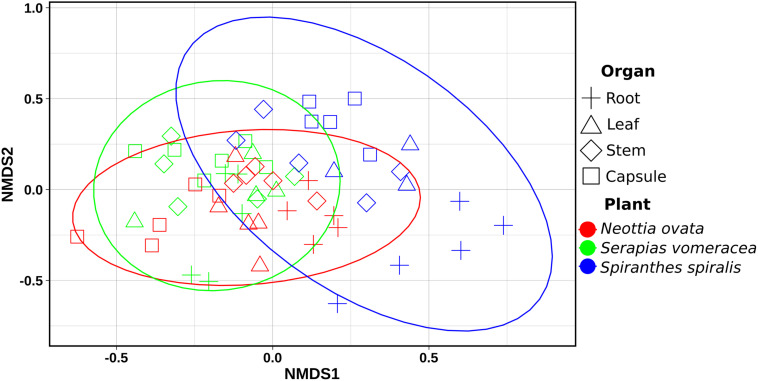
Non-metric Multidimensional Scaling (NMDS) Plot visualization of beta diversity, using the Bray–Curtis distances, separating samples by orchid species/organs. Plot ellipses represent the 95% confidence regions for species clusters (stress = 0.24).

Non-metric multidimensional scaling (NMDS) ordination analysis showed a large overlap in the microbiota of *S. vomeracea* and *N. ovata*, whereas the *S. spiralis* samples, in particular the root samples, clustered separately (Figure 5). These results were confirmed by ADONIS pairwise comparisons of microbiota structure between orchid species and organs (Table 2). Although the homogeneity of the sample dispersion was reduced in the species groups, *S. spiralis* was significantly different from *N. ovata* and *S. vomeracea*. The sample dispersion was heterogeneous in the organ groups, which showed significant differences except for the stems-leaves comparison (Table 2). Analysis of Alpha- and beta-diversity was also performed without considering the different number of sequences per sample (non-rarefied dataset). The respective results showed no substantial differences with those obtained with the rarefied datasets (compare Supplementary Figure 2 with Figure 4, Supplementary Figure 3 with Supplementary Figure 1, Supplementary Figure 4 with Figure 5, and Supplementary Table 5 with Table 2).

**TABLE 2:** ADONIS pairwise comparisons of microbiota structure between orchid species and organs, calculated on Bray–Curtis dissimilarity measures.

Species comparison	ADONIS Test	
	R^2^	*p*-value
*Serapias vomeracea* vs. *Neottia ovata*	0.0458	0.0608
*Serapias vomeracea* vs. *Spiranthes spiralis*	0.1023	**0.0002**
*Neottia ovata* vs. *Spiranthes spiralis*	0.0945	**0.0002**
**Organ comparison**		
Roots vs. Stems	0.0831	**0.0049**
Roots vs. Leaves	0.0649	**0.0436**
Roots vs. Capsules	0.0897	**0.0010**
Stems vs. Leaves	0.0528	0.1304
Stems vs. Capsules	0.1140	**0.0006**
Leaves vs. Capsules	0.0897	**0.0064**

*Significant differences are highlighted in bold.*

### Core Microbiota in the Three Orchid Species

**FIGURE 6:**
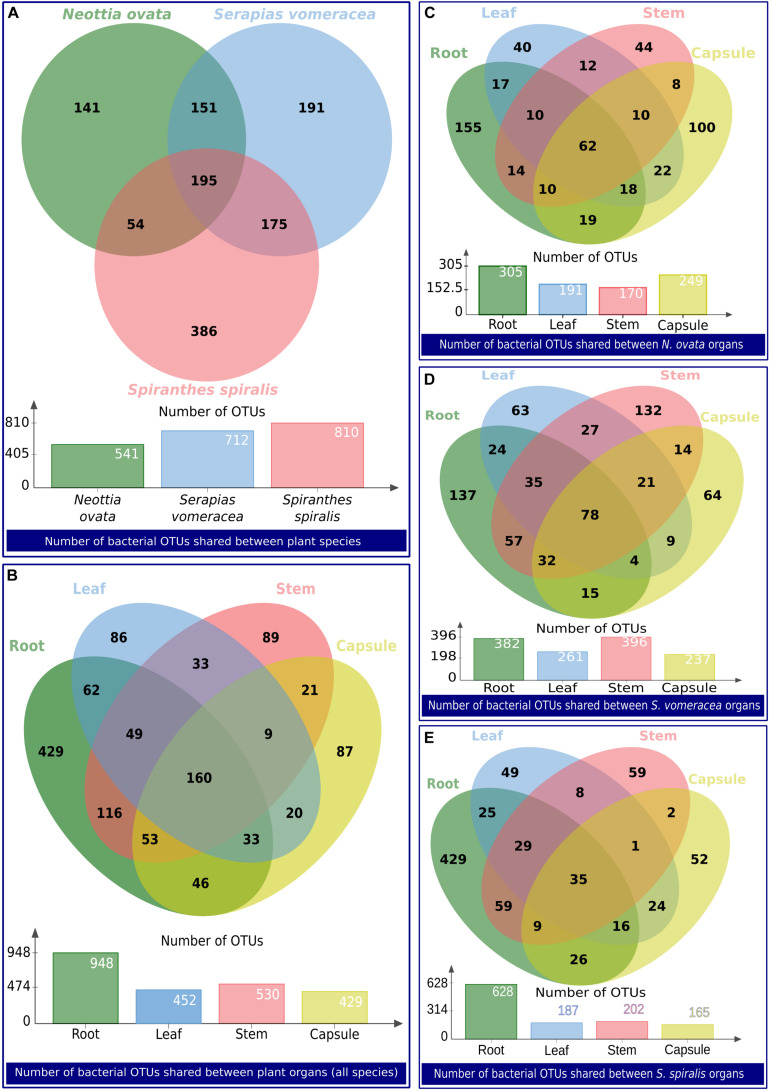
Venn diagrams showing the number of bacterial OTUs shared between groups of samples. The groups of samples are indicated as a label below each diagram.

The numbers of unique and shared OTUs identified in the orchid species and/or organs are indicated in the Venn diagrams in Figure 6. Overall, 718 OTUs were unique to *N. ovata*, *S. vomeracea* and *S. spiralis* (141, 191, and 386, respectively), whereas 195 OTUs were shared between all three species (Figure 6A); the latter represent the “species core microbiota.” When the organs of all species were considered, 691 OTUs were found to be unique to roots, stems, leaves and capsules (429, 89, 86, and 87 OTUs, respectively), 298 and 144 were shared between two and three organs, respectively, and 160 were shared by all orchid organs (Figure 6B); the latter represent the “organ core microbiota.” Considering the microbiota of the different organs in each orchid species, the numbers of unique and shared OTUs are indicated for *N. ovata* (Figure 6C), *S. vomeracea* (Figure 6D) and *S. spiralis* (Figure 6E). These shared OTUs corresponded to a similar percentage of the total identified OTUs for *N. ovata* (11.46%) and *S. vomeracea* (10.95%), whereas a lower percentage (4.25%) was found in *S. spiralis*.

Shared OTUs were taxonomically diversified (Supplementary Table 6), and no dominant phyla could be identified: relative frequencies reflected those of the total community (Figure 2). Mainly plant-associated genera formed the core microbiota of the three orchid species, also including typically beneficial taxa, such as *Agromyces*, *Bacillus*, *Bradyrhizobium*, *Burkholderia*, *Comamonas*, *Luteibacter*, *Methylobacterium*, *Pseudomonas*, *Rhizobium*, *Sphingomonas*, *Stenotrophomonas*, and *Variovorax*, among others (Supplementary Table 6). Further taxa were members of *Enterobacteriaceae* as well as other typically human-associated taxa (*Cutibacterium*, *Escherichia*-*Shigella*, *Neisseria*, and *Staphylococcus*). Typical phytopathogens were absent.

### Co-occurrence Network Analysis

**FIGURE 7:**
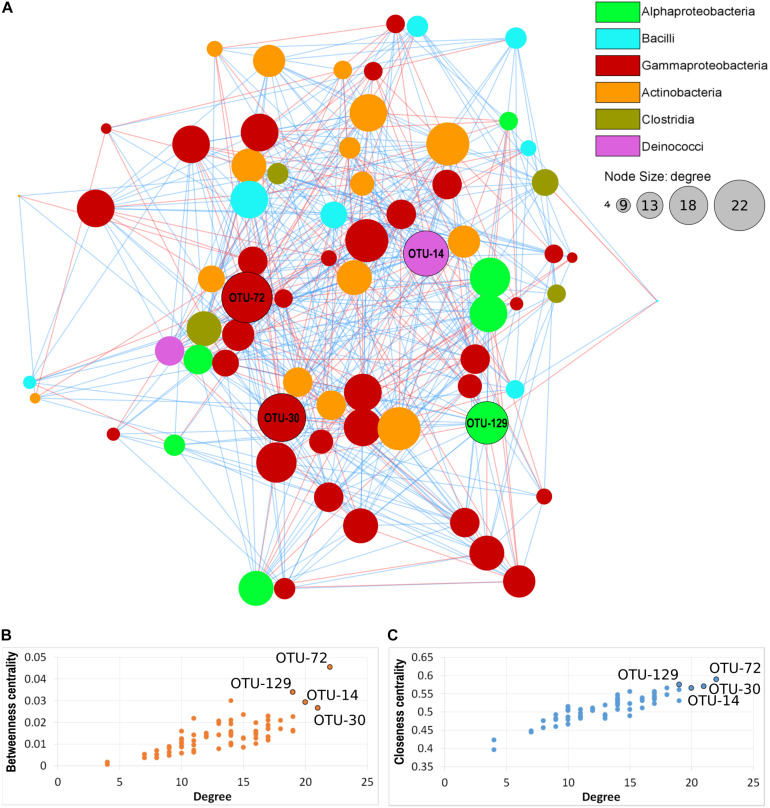
Microbial co-occurrence network of the orchid microbiota. **(A)** Nodes represent OTUs, edges represent positive (blue lines) or negative (red lines) correlations. Node size indicates the degree (number of connections) and node color indicate the taxonomical class, according to the legend. Labeled nodes represent the hub OTUs. **(B,C)** Connectivity scores of the nodes, based on degree, betweenness centrality and closeness centrality, used to identify the hub OTUs (labeled nodes).

Seventy nodes (corresponding to OTUs) composed the bacterial interaction network; each node showed 4–22 edges (significant correlations). In total, there were 465 correlations (345 positives and 120 negative). Seventeen correlations were significant for three of the four distance/correlation methods calculated, while 448 were significant for all four methods. The network was not modular (Figure 7A), thus indicating a relative homogeneity of the microbiota across orchid species and organs. Based on connectivity scores (Figures 7B,C), four OTUs were identified as “hub taxa” (Figure 7, labeled nodes). After manual BLAST, they were identified at the genus level as *Mitsuaria*, *Pseudoxanthomonas*, *Thermus*, and *Rhizobium*. Three species have been described in the genus *Mitsuaria*, and the phylogenetic analysis of OTU72 showed that it was closer to *M. noduli* and *M. chitinosanitabida* than to the recently described *M. chitinovorans* (Sisinthy and Gundlapally, 2020; Supplementary Figure 5).

Three of the hub OTUs had more positive than negative correlations, while OTU-72 (*Mitsuaria*) had the same number (11) of positive and negative correlations. These hub OTUs represented 0.04, 0.37, 0.54, and 2.62 (*Rhizobium*, *Pseudoxanthomonas*, *Mitsuaria*, and *Thermus*, respectively) of the total microbiota. *Mitsuaria* and *Thermus* were included in the core microbiota of all species, whereas *Pseudoxanthomonas* and *Rhizobium* were included in the core of *S. vomeracea* and *S. spiralis*, respectively.

### Occurrence of Endophytic Bacteria in Seeds of *Spiranthes spiralis*

**FIGURE 8:**
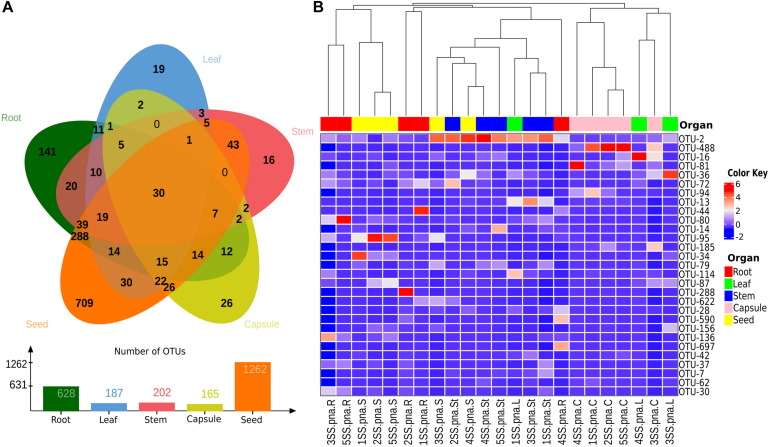
Analyses of shared and major bacterial OTUs in the *Spiranthes spiralis* microbiota. **(A)** Venn diagram showing unique and shared OTUs between vegetative and reproductive organs (root, leaf, stem, capsule, seed). **(B)** Heatmap showing bacterial OTUs > 0.5% of the total sequences; samples were clustered by orchid organs (top row). Color Key from blue to red indicates increasing abundance in natural logarithm of counts, with OTUs along the Y-axis and samples along X-axis.

To investigate the possibility of a vertical transmission of bacterial components of the orchid microbiota, seeds were obtained from surface sterilized *S. spiralis* capsules, hatched inside sterile falcon tubes. Quite surprisingly, metabarcoding of the seed microbiota yielded the highest number of OTUs (1,262), when compared with the other organs of *S. spiralis* (Figure 8). Overall, 911 OTUs were unique to the roots, stems, leaves, capsules, and seeds (141, 16, 19, 26, and 709, respectively), whereas 30 OTUs represented the core microbiota of *S. spiralis*. Interestingly, the seeds shared a notably higher number of OTUs with the roots (288) than with other organs (43 with stems, 30 with leaves and 26 with capsules).

## Discussion

Because of their dependence on specific biotic interactions, aboveground with pollinators and belowground with mycorrhizal fungi, orchids may be predisposed to become endangered, especially in a scenario of global climate change (Dearnaley et al., 2012). In addition to these well-studied interactions, orchids also establish multiple interactions with a pervasive plant microbiota that colonizes the interior of both below- and aboveground tissues. Endophytic bacteria likely play important functions for their host, and many bacterial endophytes isolated from orchid tissues showed plant growth promoting potential. For example, endophytic bacteria isolated from the tropical orchid *Dendrobium nobile* could promote orchid seed germination (Tsavkelova et al., 2016), and bacterial strains isolated from *Cymbidium* sp. could promote orchid acclimatization and/or growth (Faria et al., 2013; Gontijo et al., 2018). Although these observations suggest important roles of the bacterial orchid microbiota, very little is known about orchid-associated microbial communities, the level of specificity of their interactions with the host, their distribution in the plant and their modes of transmission across individuals and generations.

Here, we have explored the association of three terrestrial orchid species with their endophytic microbiota. Our analysis targeted the different taxonomic groups of bacteria that populated both vegetative and reproductive orchid organs, which, to the best of our knowledge, have not yet been studied in depth. By using an NGS-based metabarcoding approach, we have revealed different taxonomic groups in the microbiota of *N. ovata*, *S. vomeracea*, and *S. spiralis*, which could potentially play key roles by interacting with the host. The results confirmed our first hypothesis that the microbiota is organized in distinctive phylogenetic structures in various plant species and organs. Analysis of alpha and beta-diversity suggested that organs were the main determinant of the bacterial composition of the microbiota in orchids. There was in fact no evidence of clustering of bacterial communities according to plant species, and *N. ovata* and *S. vomeracea* were highly similar despite the very different habitat they colonize. *N. ovata* was collected in woods, whereas *S. vomeracea* is a meadow orchid species that was collected on poor grassland, a habitat more similar to the one supporting *S. spiralis* growth. This is in contrast to studies performed on different *Agave* species (Coleman-Derr et al., 2016) but is coherent with a recent study on the diversity patterns of endophytic bacterial communities of three rainforest plant species, where an extensive overlap in bacterial communities was observed between different plant organs and plant species (Haruna et al., 2018). Significant differences between plant organs were actually detected in our orchids, with a clear trend of decreasing diversity and richness along the vertical axis, that became significant when the two extremes (capsules and roots) were compared. This situation suggests an active role of the orchid plants in selecting specific bacterial taxa, more and more specialized while moving from the below-ground to the aerial parts. Bacterial root endophytes likely derive from the soil and rhizosphere microbiota (Compant et al., 2010). Once they enter the root tissues, bacterial endophytes can spread and colonize aerial plant parts, although endophytic bacteria can also enter the leaves through the stomata (Senthilkumar et al., 2011).

Endophytic bacteria in plant organs are not randomly distributed, and some dominant phyla appear to be tissue- or organ-specific. Numerous studies, conducted with different plant organs, revealed significant differences in the composition of the plant endophytic microbiota (Bodenhausen et al., 2013; Romero et al., 2014; Akinsanya et al., 2015; Maropola et al., 2015; Sarria-Guzmán et al., 2016; Li et al., 2017; Tian and Zhang, 2017; Yang et al., 2017). In the three orchid species, Proteobacteria and Actinobacteria were the dominant groups, irrespective of the plant species, but with differences in the distribution in the plant organs. In addition, the distribution of the bacterial genera did not determine a clear clustering by species/organ. This suggests a good overlap of the key members of the community in the different orchid species, some of which could be involved in essential processes, such as nitrogen fixation (Sun et al., 2004; Moyes et al., 2016; Puri et al., 2016).

Among the most representative genera, *Cutibacterium* has been identified, which is widely known as a commensal and is part of the human skin microbiota. However, a recent study, in support of our data, demonstrated the presence of *Cutibacterium* as an endophyte of roots and leaves in the wheat cultivars *Triticum spelta* cv Rokosz and *Triticum aestivum* cv Hondia (Kuźniar et al., 2020). It is also very interesting, as reported by Shaffer et al. (2018), that this genus has been classified as a hyphal endosymbiont of a leaf fungal endophyte (*Fusarium concolor*) from *Hybanthus prunifolius*. This aspect deserves to be deepened by further studies to understand the functional roles that could possibly mediate tripartite interactions with orchid mycorrhizal fungi.

The other most representative genera, distributed differently in the three orchid species, were *Pseudomonas*, *Luteibacter*, *Cupriavidus, Limnobacter, Pantoea*, and *Acinetobacter* which are included among the genera most commonly found as bacterial endophytes (Marquez-Santacruz et al., 2010; Romero et al., 2014; Shi et al., 2014). *Pseudomonas* species, thanks to their metabolic versatility and ubiquity, colonize several natural habitats by adopting a variety of lifestyles. This genus has been identified as an endosymbiont group in Australian terrestrial orchids (Wilkinson et al., 1989, 1994), and in the roots of epiphytic orchids (Tsavkelova et al., 2004).

The genus *Pantoea* is a diverse group of bacteria commonly found in association with plants. *Pantoea* endophytes were found mainly in plants such as vine, pea, rice and barley (Bell et al., 1995; Elvira-Recuenco and van Vuurde, 2000; Mano et al., 2007; Rahman et al., 2018).

Their potential role as PGP for host plants is well documented, such as ACC-deaminase activity (Zhang et al., 2011), hormone production (Tsavkelova et al., 2007; Chalupowicz et al., 2009), and siderophores (Loaces et al., 2011). As demonstrated by our recent study (Alibrandi et al., 2020), endophytes isolated from the same orchid species investigated in the current work, belonging to *Pseudomonas* and *Pantoea* genera, can use different mechanisms to promote plant growth. Strains classified in the genus *Luteibacter* were found in close association with plants (Johansen et al., 2005; Aamot et al., 2017) and lichens (Cardinale et al., 2006). In *Luteibacter* genus, some strains were also characterized as endohyphal bacteria within a range of ascomycete fungi (Arendt et al., 2016; Araldi-Brondolo et al., 2017). These bacterial / fungal complexes modulate several fungal phenotypes, including the possibility that some fungi act as plant growth promoters (Arendt et al., 2016; Araldi-Brondolo et al., 2017). This genus was mainly distributed in the two orchid species (*N. ovata* and *S. vomeracea*) that showed no significant differences in microbiota structure, suggesting a species-dependent colonization for this genus.

Members of the *Limnobacter* genus have often been detected in various environments, such as surface seawater, deep ocean, human intestine and volcanic deposits (Lu et al., 2008; Eloe et al., 2010; Rigsbee et al., 2011; Vedler et al., 2013). However, *Limnobacter* was found in maize seeds (cultivar Ngonda 108) at different stages of growth (Liu et al., 2013). We have identified *Limnobacter* as the dominant genus in *N. ovata* capsules, indicating that these bacteria tend to colonize preferentially the aerial parts of the orchids.

*Cupriavidus* species have been commonly isolated in different habitats from soil, water, plant nodules and human medical specimens (Coenye et al., 1999, 2003; Chen et al., 2001; Goris et al., 2001; Sato et al., 2006). It is known that some strains isolated from different mimosa species growing in distinct regions of China, Costa Rica, Taiwan, and Papua New Guinea (Chen et al., 2003; Barrett and Parker, 2006; Elliott et al., 2009; Liu et al., 2011) induce the formation of root nodules in legume plants. The *Cupriavidus* genus was found mainly in *N. ovata* and *S. vomeracea* (more abundant in the stems) and was distributed in both above- and below-ground organs. This result suggests that members of this genus are able to colonize the whole plant regardless of the organ.

*Acinetobacter* includes heterogeneous bacteria, typically free-living saprophytes and ubiquitous, associated with various habitats, e.g., soil, water, wastewater, humans, used kitchen sponges, food and animals (Visca et al., 2011; Doughari et al., 2011; Jung and Park, 2015; Cardinale et al., 2017). *Acinetobacter* is also known as an endophytic bacterium and plant growth promoter. *A. calcoaceticus* strains isolated from coffee plant tissues were capable of producing phosphatases and siderophores (Silva et al., 2012). Similarly, *A. johnsonii* isolated from surface-sterilized roots of *Beta vulgaris* showed the ability to increase the absorption of N, P, K, and Mg and produced the phytohormone auxin. Through these traits, this PGP strain was able to promote the growth of sugar beet by increasing the yield of sucrose and fructose (Shi et al., 2011). We found the *Acinetobacter* genus distributed in all organs of *S. spiralis*, which indicates a species preference for colonization independently of the plant organ.

Co-occurrence analysis identified four hub OTUs, two of them being typical plant-associated taxa, namely *Pseudoxanthomonas* and *Rhizobium*. *Rhizobium* typically forms nitrogen fixing symbioses with legume roots but is also able to promote plant growth in non-legume plants (García-Fraile et al., 2012). *Mitsuaria* is a genus within the Betaproteobacteriales that includes Gram-negative, obligate aerobic, oxidase, and catalase positive bacteria (Amakata et al., 2005). To date, only three species have been identified: *M. chitosanitabida*, isolated from soil (Amakata et al., 2005), *M. noduli*, isolated from root nodules of plants growing in soils contaminated with heavy metals (Fan et al., 2018), and very recently *M. chitinivorans* isolated from tube well water (Sisinthy and Gundlapally, 2020). *Mitsuaria* isolates have been also identified as plant-associated microbes both in the rhizosphere and as plant endophytes of both monocotyledons and dicotyledons (Nascimento et al., 2015; Huang et al., 2017; Fan et al., 2018). *Mitsuaria* has been reported to have biocontrol effects against phytopathogens, such as *Rhizoctonia solani* and *Pythium aphanidermatum* in tomato and soybean (Benítez et al., 2009). This ability may relate to the unique chitinase and chitosanase that are characteristics of *Mitsuaria* (Sisinthy and Gundlapally, 2020). A suppressive effect of *Mitsuaria* species was also demonstrated against bacterial diseases caused by *Ralstonia pseudosolanacearum* (Marian et al., 2018) and drought stress in *Arabidopsis thaliana* (Huang et al., 2017). It is worth noting that *Mitsuaria* is part of the core microbiota of all three orchid species tested (Supplementary Table 6); the multiple relationships of the genus *Mitsuaria* with the other members of the orchid microbiota could generate additional synergistic effects for the benefit of the host, as recently suggested in a study on tomato plants (Marian et al., 2019).

*Thermus* is a genus of Gram-negative thermophilic bacteria belonging to the phylum *Deinococcus*-*Thermus*. As the name suggests, one of its peculiar characteristics is the ability to live in environments with extreme temperatures. *Thermus*-like bacteria have been found in both shallow and deep-sea marine hydrothermal systems, as well as in low saline sulfate sources (Huber and Stetter, 2004). It was therefore surprising to find it as an orchid endophyte, and more as a hub taxon. To date, there are no studies of *Thermus* bacteria-plant interaction, and therefore this aspect deserves attention for future studies.

On the one hand, the core microbiota included the most abundant OTUs, most of which were related to plant-beneficial taxa. However, it was interesting that the OTUs shared between roots and other organs concerned only less abundant OTUs, suggesting an important role also for the less abundant bacteria. Some microbial taxa that occur in low abundance are called “satellite taxa” (Hanski, 1982; Magurran and Henderson, 2003) and can be defined mainly based on their local abundance and specificity to the habitat (Jousset et al., 2017). It was recently suggested that taxa occurring in low abundance could manage to counteract unwanted microbial invasions in soil communities (Mallon et al., 2015) and largely contributed to the production of volatile antifungal compounds that finally protected plants from soil borne phytopathogens (Hol et al., 2015).

Seeds can host the microbiota potentially inheritable by the next plant generation, thus restoring the microbiota imprinting from the parental plants. The seed microbiota has attracted increasing interest in the last years. Seed-associated bacteria have been detected in seeds of various crop species, including rice (Bacilio-Jiménez et al., 2001; Cottyn et al., 2001; Hardoim et al., 2012), maize (Liu et al., 2013), tobacco (Mastretta et al., 2009), coffee (Vega et al., 2005), quinoa (Pitzschke, 2016), common bean (López-López et al., 2010), grapevine (Compant et al., 2011), barley (Rahman et al., 2018), pumpkin (Fürnkranz et al., 2012) and alfalfa (Charkowski et al., 2001), as well as in wild plants such as cardon cactus (*Pachycereus pringlei*) (Puente et al., 2009), *Eucalyptus* spp. (Ferreira et al., 2008), Norwegian spruce (*Picea abies*) (Cankar et al., 2005) and the South American tree *Anadenanthera colubrina* (Alibrandi et al., 2018). Surprisingly, the analysis of the *S. spiralis* seed microbiota showed that these reproductive organs were the niche of the highest number of OTUs, about twice that of the roots (organs usually associated with a greater microbial diversity). This finding contradicts several studies, which suggested that seed endophytic microbiota tends to be paucispecific, generally limited to few genera, mainly *Bacillus*, *Pseudomonas*, *Paenibacillus*, *Micrococcus*, *Staphylococcus*, *Pantoea*, and *Acinetobacter* (Truyens et al., 2015). Despite showing the highest microbiota diversity, the *S. spiralis* seeds further filtered the microbiota core (from 35 to 30 OTUs) and confirmed our second hypothesis that the seeds may represent a reservoir of endophytes that can be vertically transmitted. The most unexpected result was the large number of OTUs shared between the seeds and the roots (Figure 8), that was much higher than the fraction of the microbiota shared between the seeds and the capsules. It was curious to find that capsules and seeds (which are inside the capsules) only shared 26 OTUs, 10 times less than those shared between roots and seeds. As these shared OTUs were not identified in the other above-ground orchid tissues, the most likely explanation is that they may originate from outside the plants. A possible hypothesis on the origin of such abundant seed microbiota in *S. spiralis* is *via* the pollen. Plant pollens are a rich source of microbes (Ambika Manirajan et al., 2016, 2018), and bacteria experimentally introduced into flowers were retrieved in the seed microbiome (Mitter et al., 2017). Therefore, pollen-associated bacteria are considered to contribute to the assemblage of the seed microbiota (Rodríguez et al., 2018). Furthermore, pollination in orchid plants does not rely on individual pollen grains, but on the so-called “pollinia” (clusters of tightly aggregated pollen grains), which might increase the microbial load at each pollination event. Another possible origin is the aerosol generated by the rain when it hits the soil (Joung et al., 2017). In this case, the aerosol droplets carrying soil microbes could reach aerial parts, adhering to the sticky flower tissues and being then collected in the flower cavities more than on other plant surfaces, where rains can flush them away rapidly (Allard et al., 2020). This interesting finding should be further investigated in other sampling sites, years and on other orchid species, to generalize whether Orchidaceae can be considered as a plant family with an abundant and root-like seed microbiome. Further studies will be also required to verify the actual vertical transmission of seed-associated microbes in orchids, as well as their role in orchid growth and health.

## Conclusion

In this study, we analyzed the total endophytic bacterial microbiota associated to three terrestrial orchid species. We found that:

- Plant organs influenced the diversity and structure of the endophytic community more than plant species.
- The systemic colonization of the orchid microbiota followed a common pattern, with a reduction of diversity from the roots to the capsules, which indicates an active role of the plant in the process of microbiome selection.
- The conspicuous diversity of the seed microbiota of *Spiranthes spiralis*, which notably overlapped with that of the roots, suggests a role of orchid seeds as a vector of microbes to restore the rhizosphere microbiome. This aspect further supports the evidence of a beneficial role of the orchid microbiota for the orchid host.

This work contributes to increase our knowledge on the diversity of the bacterial microbiota of terrestrial orchids, opening new hitherto unexplored scenarios for orchid holobiont studies.

## Data Availability Statement

The datasets presented in this study can be found in online repositories. The names of the repository/repositories and accession number(s) can be found below: https://www.ebi.ac.uk/ena, PRJEB40289.

## Author Contributions

PA performed the experiments, analyzed the data, and wrote the manuscript. SS contributed to the experiments and data interpretation, and critically reviewed the manuscript. SP conceived the study, supervised the experiments, validated the data, and wrote and critically reviewed the manuscript. MC conceived the study, supervised the experiments and the data analysis, and wrote and critically reviewed the manuscript. All authors contributed to the article and approved the submitted version.

## Conflict of Interest

The authors declare that the research was conducted in the absence of any commercial or financial relationships that could be construed as a potential conflict of interest.

## Footnotes

1 https://www.phylo.org/

2 https://itol.embl.de/

3 http://bioinformatics.psb.ugent.be/webtools/Venn/

4 http://apps.cytoscape.org/apps/legendcreator
