# Supplementary material for: Diversity and Structure of the Endophytic Bacterial Communities Associated With Three Terrestrial Orchid Species as Revealed by 16S rRNA Gene Metabarcoding
Source: Front Microbiol. 2020 Dec 15;11:604964. doi: 10.3389/fmicb.2020.604964 (PMC7839077; doi:10.3389/fmicb.2020.604964)
Supplement: Supplementary file 1 [file Data_Sheet_1.doc]

**Table S1** Sequence read counts in the investigated samples, before (raw) and after filtering, as obtained by 16S rRNA metabarcoding and QIIME analysis. In red, samples eliminated due to the low number of sequence reads.

| **Sample name** | **Sample type** | **N. filtered reads^a^** | **N. filtered reads^b^** |
| --- | --- | --- | --- |
| 1NO.pna.R | *Neottia ovata* root | 14872 | 3712 |
| 2NO.pna.R | *Neottia ovata* root | 81525 | 10485 |
| 3NO.pna.R | *Neottia ovata* root | 230434 | 21921 |
| 4NO.pna.R | *Neottia ovata* root | 84467 | 43085 |
| 5NO.pna.R | *Neottia ovata* root | 123424 | 68985 |
| 1NO.pna.L | *Neottia ovata* leaf | 124733 | 7724 |
| 2NO.pna.L | *Neottia ovata* leaf | 139196 | 31770 |
| 3NO.pna.L | *Neottia ovata* leaf | 176719 | 13165 |
| 4NO.pna.L | *Neottia ovata* leaf | 79590 | 3665 |
| 5NO.pna.L | *Neottia ovata* leaf | 121435 | 12395 |
| 1NO.pna.St | *Neottia ovata* stem | 106355 | 12442 |
| 2NO.pna.St | *Neottia ovata* stem | 138309 | 21341 |
| 3NO.pna.St | *Neottia ovata* stem | 83306 | 47598 |
| 4NO.pna.St | *Neottia ovata* stem | 94665 | 19497 |
| 5NO.pna.St | *Neottia ovata* stem | 71061 | 14105 |
| 1NO.pna.C | *Neottia ovata* capsule | 132789 | 67383 |
| 2NO.pna.C | *Neottia ovata* capsule | 131636 | 52608 |
| 3NO.pna.C | *Neottia ovata* capsule | 93911 | 86523 |
| 4NO.pna.C | *Neottia ovata* capsule | 178654 | 2965 |
| 5NO.pna.C | *Neottia ovata* capsule | 91492 | 44615 |
| 1SV.pna.R | *Serapias vomeracea* root | 158718 | 154719 |
| 2SV.pna.R | *Serapias vomeracea* root | 49113 | 45852 |
| 3SV.pna.R | *Serapias vomeracea* root | 140623 | 135183 |
| 4SV.pna.R | *Serapias vomeracea* root | 97402 | 96791 |
| 5SV.pna.R | *Serapias vomeracea* root | 39425 | 36689 |
| 1SV.pna.L | *Serapias vomeracea* leaf | 68541 | 10784 |
| 2SV.pna.L | *Serapias vomeracea* leaf | 169618 | 106852 |
| 3SV.pna.L | *Serapias vomeracea* leaf | 67478 | 1617 |
| 4SV.pna.L | *Serapias vomeracea* leaf | 49553 | 16118 |
| 5SV.pna.L | *Serapias vomeracea* leaf | 17280 | 856 |
| 1SV.pna.St | *Serapias vomeracea* stem | 43027 | 7689 |
| 2SV.pna.St | *Serapias vomeracea* stem | 15882 | 8866 |
| 3SV.pna.St | *Serapias vomeracea* stem | 21545 | 17164 |
| 4SV.pna.St | *Serapias vomeracea* stem | 49761 | 20429 |
| 5SV.pna.St | *Serapias vomeracea* stem | 35442 | 17694 |
| 1SV.pna.C | *Serapias vomeracea* capsule | 76723 | 3075 |
| 2SV.pna.C | *Serapias vomeracea* capsule | 96062 | 3419 |
| 3SV.pna.C | *Serapias vomeracea* capsule | 75003 | 40828 |
| 4SV.pna.C | *Serapias vomeracea* capsule | 73642 | 5860 |
| 5SV.pna.C | *Serapias vomeracea* capsule | 32634 | 2154 |
| 1SS.pna.R | *Spiranthes spiralis* root | 26237 | 392 |
| 2SS.pna.R | *Spiranthes spiralis* root | 24966 | 6458 |
| 3SS.pna.R | *Spiranthes spiralis* root | 49762 | 8158 |
| 4SS.pna.R | *Spiranthes spiralis* root | 17117 | 239 |
| 5SS.pna.R | *Spiranthes spiralis* root | 1668 | 1190 |
| 1SS.pna.L | *Spiranthes spiralis* leaf | 9271 | 225 |
| 2SS.pna.L | *Spiranthes spiralis* leaf | 3 | 3 |
| 3SS.pna.L | *Spiranthes spiralis* leaf | 2791 | 307 |
| 4SS.pna.L | *Spiranthes spiralis* leaf | 22164 | 6356 |
| 5SS.pna.L | *Spiranthes spiralis* leaf | 909 | 74 |
| 1SS.pna.St | *Spiranthes spiralis* stem | 533 | 221 |
| 2SS.pna.St | *Spiranthes spiralis* stem | 22730 | 6737 |
| 3SS.pna.St | *Spiranthes spiralis* stem | 24983 | 913 |
| 4SS.pna.St | *Spiranthes spiralis* stem | 24149 | 612 |
| 5SS.pna.St | *Spiranthes spiralis* stem | 34074 | 28583 |
| 1SS.pna.C | *Spiranthes spiralis* capsule | 2758 | 1609 |
| 2SS.pna.C | *Spiranthes spiralis* capsule | 1018 | 748 |
| 3SS.pna.C | *Spiranthes spiralis* capsule | 1411 | 1174 |
| 4SS.pna.C | *Spiranthes spiralis* capsule | 12689 | 11217 |
| 5SS.pna.C | *Spiranthes spiralis* capsule | 6385 | 4224 |
| 1SS.pna.S | *Spiranthes spiralis* seed | 28382 | 6509 |
| 2SS.pna.S | *Spiranthes spiralis* seed | 15571 | 10351 |
| 3SS.pna.S | *Spiranthes spiralis* seed | 22338 | 4461 |
| 4SS.pna.S | *Spiranthes spiralis* seed | 12949 | 2348 |
| 5SS.pna.S | *Spiranthes spiralis* seed | 76735 | 47468 |
| **Total** |  | 4,117,638 | 1,469,200 |

^a^ after quality/length filtering

^b^ after contaminant sequences filtering (mitochondrial, plastid and unassigned OTUs)

**Table S2** Alpha diversity estimation of richness (observed OTUs), diversity (Shannon), Phylogenetic diversity (PD) and Good’s coverage of *Neottia ovata* samples microbiota, as assessed by 16S rRNA gene metabarcoding and calculated on the dataset rarefied at 2965 sequences per sample

| **Sample** | **Observed OTUs** | **Shannon** | **PD** | **Good’s coverage** |
| --- | --- | --- | --- | --- |
| 1NO.pna.R | 70 | 3.75 | 9.94 | 1.00 |
| 2NO.pna.R | 84 | 3.62 | 11.07 | 0.99 |
| 3NO.pna.R | 70 | 3.18 | 9.13 | 1.00 |
| 4NO.pna.R | 64 | 3.56 | 9.50 | 0.99 |
| 5NO.pna.R | 94 | 4.35 | 10.58 | 0.99 |
| 1NO.pna.L | 58 | 3.75 | 8.40 | 1.00 |
| 2NO.pna.L | 60 | 3.97 | 5.61 | 0.99 |
| 3NO.pna.L | 62 | 3.95 | 9.11 | 1.00 |
| 4NO.pna.L | 48 | 3.82 | 3.96 | 1.00 |
| 5NO.pna.L | 52 | 2.66 | 7.96 | 1.00 |
| 1NO.pna.St | 48 | 3.22 | 4.78 | 1.00 |
| 2NO.pna.St | 41 | 3.19 | 6.59 | 1.00 |
| 3NO.pna.St | 49 | 3.47 | 7.23 | 1.00 |
| 4NO.pna.St | 55 | 3.81 | 5.07 | 1.00 |
| 5NO.pna.St | 58 | 3.87 | 8.34 | 1.00 |
| 1NO.pna.C | 78 | 3.40 | 9.70 | 0.99 |
| 2NO.pna.C | 43 | 2.68 | 7.56 | 0.99 |
| 3NO.pna.C | 26 | 0.91 | 2.97 | 1.00 |
| 4NO.pna.C | 66 | 3.21 | 8.98 | 0.99 |

**Table S3** Alpha diversity estimation of richness (observed species), diversity (Shannon), Phylogenetic diversity (PD) and Good’s coverage of *Serapias vomeracea* samples microbiota, as assessed by 16S rRNA gene metabarcoding and calculated on the dataset rarefied at 1617 sequences per sample

| **Sample** | **Observed OTUs** | **Shannon** | **PD** | **Good’s coverage** |
| --- | --- | --- | --- | --- |
| 1SV.pna.R | 69 | 3.50 | 9.61 | 0.96 |
| 2SV.pna.R | 46 | 2.69 | 6.82 | 0.97 |
| 3SV.pna.R | 54 | 3.08 | 5.78 | 0.98 |
| 4SV.pna.R | 31 | 3.37 | 3.83 | 0.99 |
| 5SV.pna.R | 47 | 2.87 | 7.99 | 0.98 |
| 1SV.pna.L | 51 | 2.66 | 8.50 | 0.98 |
| 2SV.pna.L | 25 | 1.97 | 5.83 | 0.98 |
| 3SV.pna.L | 52 | 4.14 | 8.54 | 0.98 |
| 4SV.pna.L | 64 | 4.37 | 9.21 | 0.98 |
| 5SV.pna.L | 32 | 2.94 | 6.93 | 0.99 |
| 1SV.pna.St | 65 | 3.75 | 9.09 | 0.98 |
| 2SV.pna.St | 52 | 2.04 | 9.06 | 0.97 |
| 3SV.pna.St | 75 | 3.81 | 9.52 | 0.96 |
| 4SV.pna.St | 67 | 4.54 | 6.14 | 0.98 |
| 5SV.pna.St | 33 | 1.86 | 3.80 | 0.99 |
| 1SV.pna.C | 27 | 2.42 | 3.25 | 0.99 |
| 2SV.pna.C | 42 | 2.69 | 8.12 | 0.98 |
| 3SV.pna.C | 29 | 1.85 | 3.24 | 0.98 |
| 4SV.pna.C | 49 | 2.99 | 5.72 | 0.97 |

**Table S4** Alpha diversity estimation of richness (observed species), diversity (Shannon), Phylogenetic diversity (PD) and Good’s coverage of *Spiranthes spiralis* samples microbiota, as assessed by 16S rRNA gene metabarcoding and calculated on the dataset rarefied at 221 sequences per sample

| **Sample** | **Observed OTUs** | **Shannon** | **PD** | **Good’s coverage** |
| --- | --- | --- | --- | --- |
| 1SS.pna.R | 29 | 3.17 | 2.43 | 0.94 |
| 2SS.pna.R | 69 | 4.96 | 10.60 | 0.82 |
| 3SS.pna.R | 102 | 6.21 | 13.45 | 0.76 |
| 4SS.pna.R | 57 | 4.87 | 6.62 | 0.88 |
| 5SS.pna.R | 68 | 5.12 | 9.56 | 0.85 |
| 1SS.pna.L | 45 | 4.06 | 8.54 | 0.88 |
| 3SS.pna.L | 49 | 4.11 | 8.55 | 0.89 |
| 4SS.pna.L | 19 | 1.64 | 5.12 | 0.95 |
| 1SS.pna.St | 66 | 5.16 | 9.47 | 0.84 |
| 2SS.pna.St | 27 | 3.35 | 3.24 | 0.96 |
| 3SS.pna.St | 45 | 3.90 | 7.86 | 0.90 |
| 4SS.pna.St | 22 | 3.04 | 2.56 | 0.97 |
| 5SS.pna.St | 23 | 2.57 | 6.52 | 0.94 |
| 1SS.pna.C | 21 | 2.37 | 5.59 | 0.96 |
| 2SS.pna.C | 19 | 2.07 | 2.56 | 0.95 |
| 3SS.pna.C | 40 | 3.94 | 7.62 | 0.90 |
| 4SS.pna.C | 14 | 1.55 | 2.33 | 0.97 |

**Table S5** ADONIS pairwise comparisons of microbiota structure between orchid species and organs, calculated on Bray-Curtis dissimilarity measures of not rarefied dataset. Significant differences are highlighted in bold.

| **Species comparison** | **ADONIS Test** | |
| --- | --- | --- |
|  | **R^2^** | **p-value** |
| *Serapias vomeracea* vs. *Neottia ovata* | 0.0306 | 0.0374 |
| *Serapias vomeracea* vs. *Spiranthes spiralis* | 0.0374 | **0.0001** |
| *Neottia ovata* vs. *Spiranthes spiralis* | 0.0364 | **0.0002** |
| **Organ comparison** |  |  |
| Roots vs. Stems | 0.0439 | **0.0114** |
| Roots vs. Leaves | 0.0465 | **0.0038** |
| Roots vs. Capsules | 0.0460 | **0.0004** |
| Stems vs. Leaves | 0.0396 | 0.1450 |
| Stems vs. Capsules | 0.0434 | **0.0005** |
| Leaves vs. Capsules | 0.0433 | **0.0098** |

**Table S6 (included as excel file)** Core microbiome OTUs detected in three terrestrial orchid species


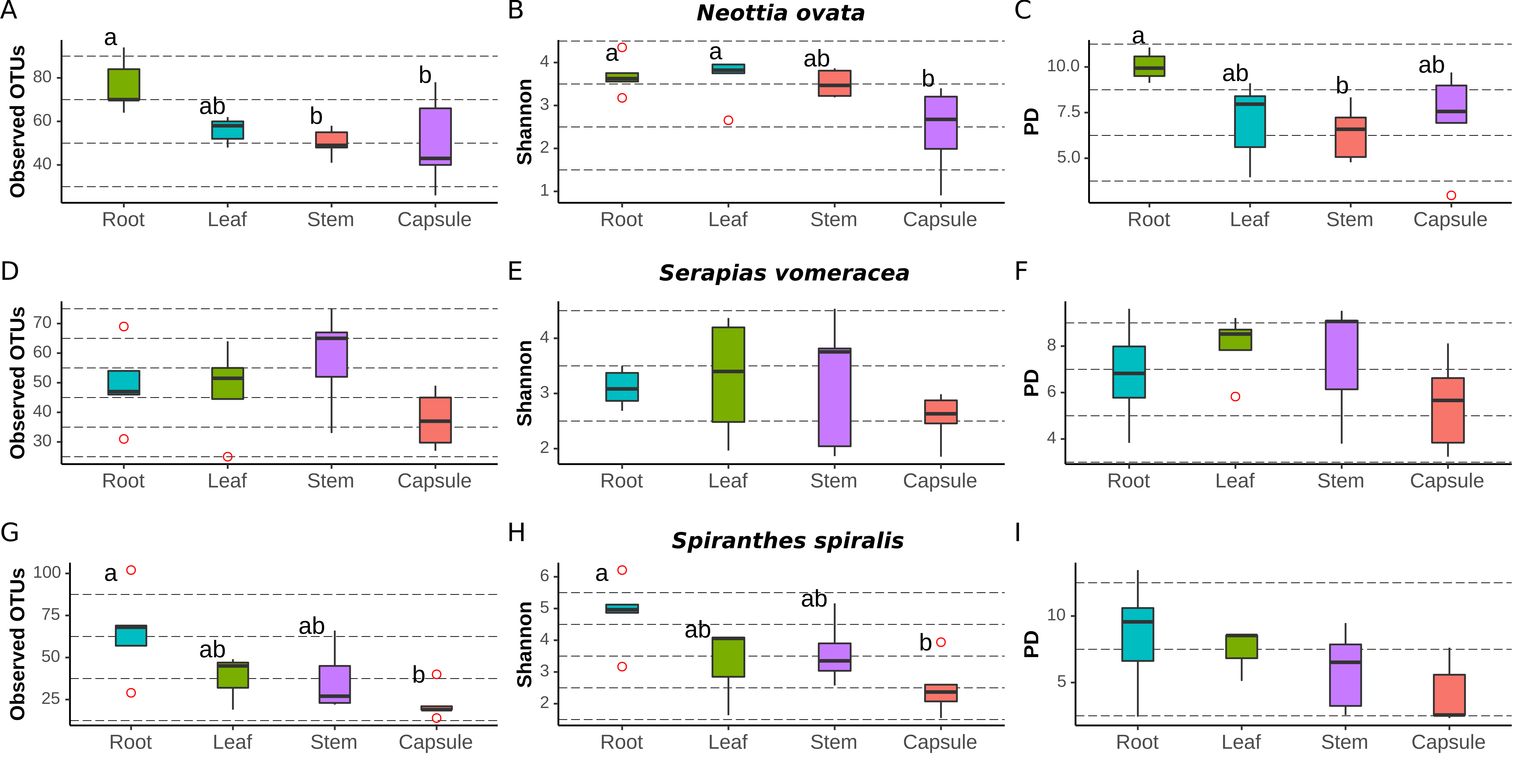


**Fig. S1** Comparison of alpha-diversity indices between organs within each orchid plant. Richness (observed OTUs), diversity (Shannon) and phylogenetic diversity (PD) were used. ° indicates outliers. Different letters above the bars indicate significantly different means (Tukey test, p < 0.05); no letters (panels D-F and I) indicate no statistically significant differences.


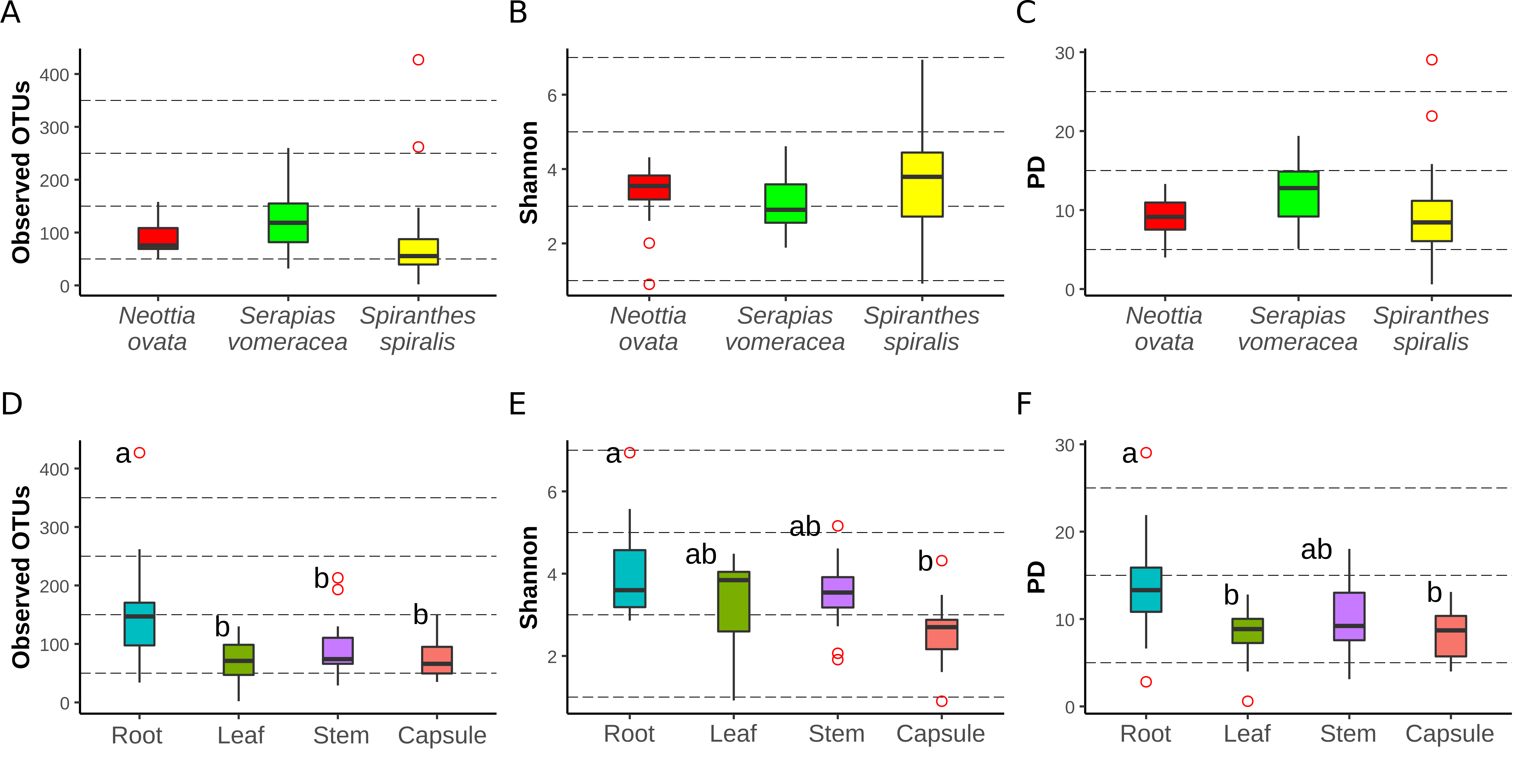


**Fig S2** Comparison of alpha-diversity metrics between the microbiota of three Mediterranean orchid species, calculated on not rarefied dataset. Richness (observed OTUs), diversity (Shannon) and phylogenetic diversity (PD) for different plant species (A-C) and organs (D-F). ° indicates outliers. Different letters above the bars indicate significantly different means (Tukey test, p < 0.05); no letters indicate no statistically significant differences.


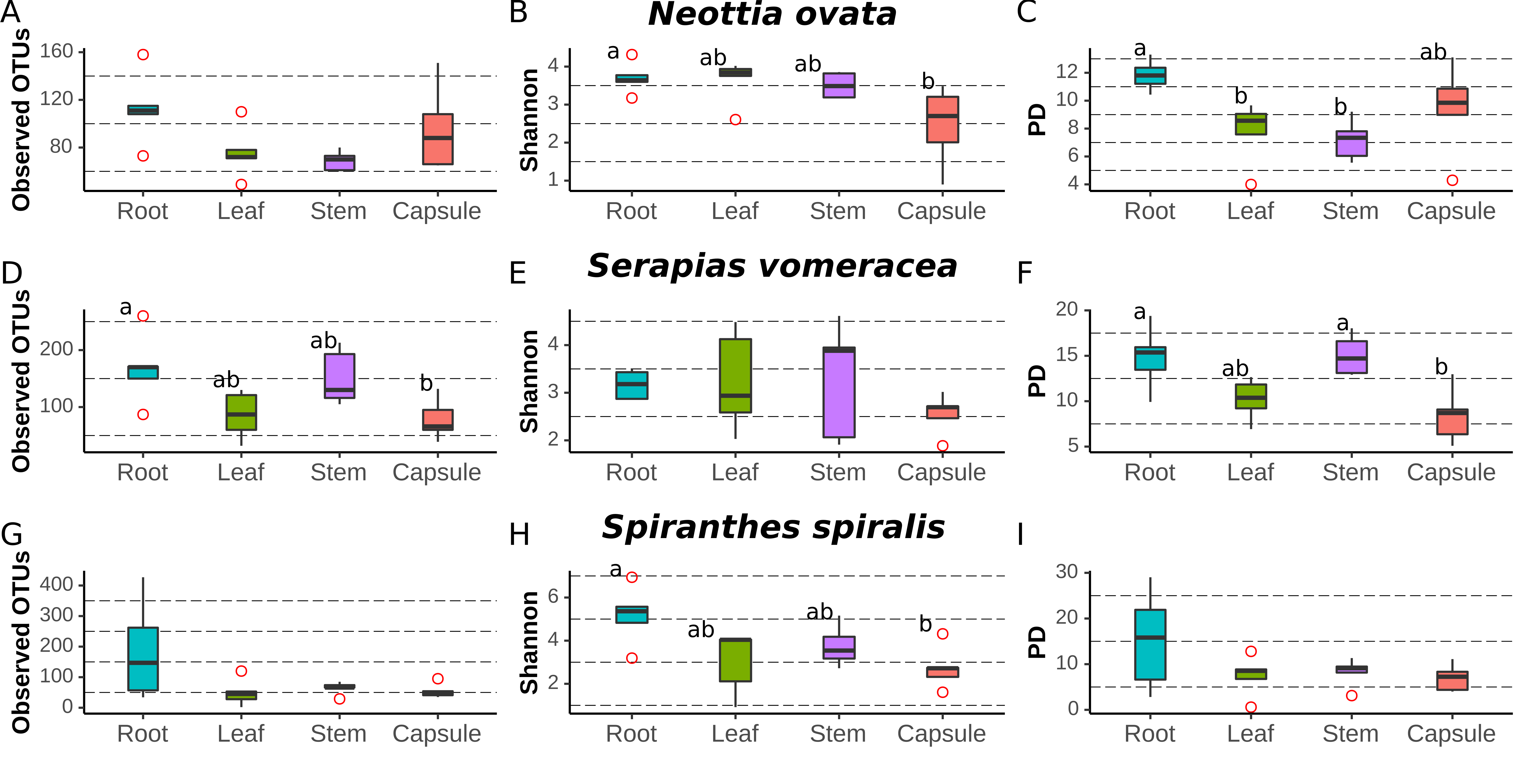
**Fig. S3** Comparison of alpha-diversity indices between the microbiota of plant organs within each orchid species, calculated on not rarefied dataset. Richness (observed OTUs), diversity (Shannon) and phylogenetic diversity (PD) were used. ° indicates outliers. Different letters above the bars indicate significantly different means (Tukey test, p < 0.05); no letters indicate no statistically significant differences.


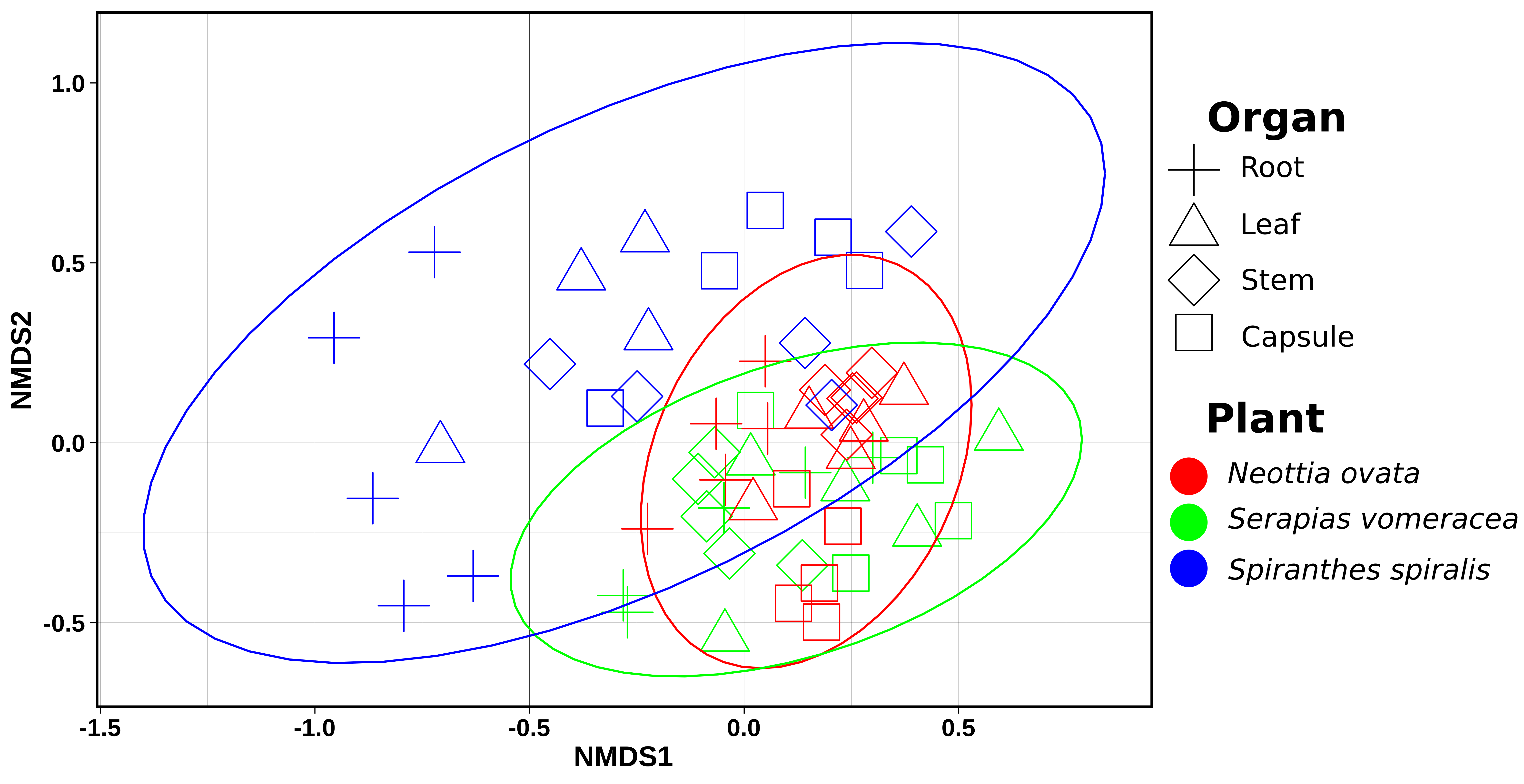


**Fig. S4** Non-metric Multidimensional Scaling (NMDS) Plot visualization of beta diversity, using the Bray–Curtis distances, separating samples by orchid species/organs of no rarefied dataset. Plot ellipses represent the 95% confidence regions for species clusters (stress= 0.24).


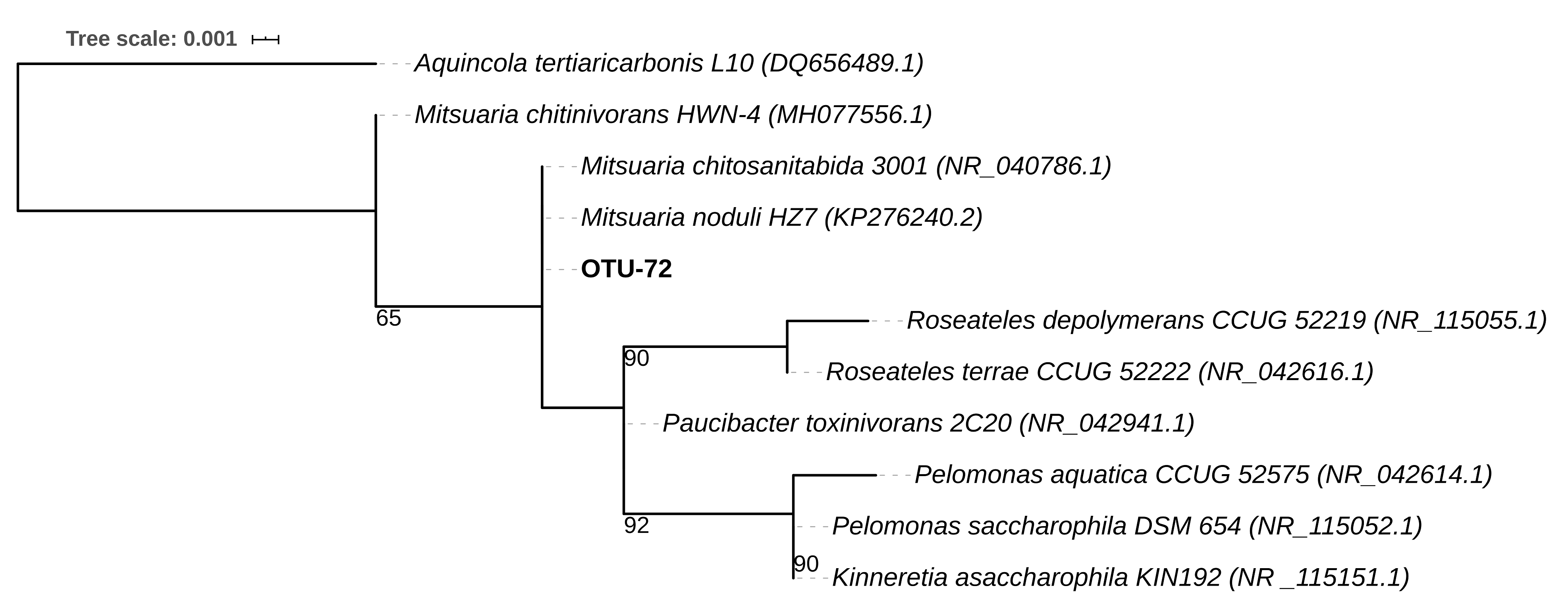


**Fig. S5** Phylogenetic tree of OTU-72, based on16S rRNA gene partial sequence. Maximum likelihood 16S rRNA gene phylogenetic tree including the ambiguous taxon sequence (bold) and 16S reference sequences of *Mitsuaria*, *Roseateles*, *Paucibacter*, *Pelomonas* and *Kinneretia* type strains. *Aquincola tertiaricarbonis* L10 (GenBank accession (DQ656489.1) was used as outgroup. This tree was generated using the RAxML method; the numbers at the branch nodes indicate the bootstrap percentage values obtained from 1000 resamplings (only bootstraps >50% are shown). Scale bar indicates substitutions per site.
